# The Impact of Obesity on Patient Reported Outcomes Following Stereotactic Body Radiation Therapy for Prostate Cancer

**DOI:** 10.7759/cureus.669

**Published:** 2016-07-05

**Authors:** Harsha Koneru, Robyn Cyr, Li Rebekah Feng, Edward Bae, Malika T Danner, Marilyn Ayoob, Thomas M Yung, Siyuan Lei, Brian T Collins, Leorey Saligan, Suy Simeng, Deepak Kumar, Sean P Collins

**Affiliations:** 1 Department of Radiation Medicine, Georgetown University Hospital; 2 National Institute of Nursing Research, National Institutes of Health; 3 National Institute of Nursing Research, National Institute of Health; 4 Deptartment of Biological & Environmental Sciences, University of the District of Columbia

**Keywords:** organ confined prostate cancer, sbrt, bmi, obesity, cyberknife, epic

## Abstract

**Objectives:**

The relationship between obesity (Body Mass Index ­>30 kg/m^2^) and quality of life (QoL) following prostate cancer (PCa) radiation therapy (RT) is unknown. Excess abdominal fat may compromise the precise delivery of radiation, putting surrounding organs at risk for greater radiation exposure. Stereotactic body radiation therapy (SBRT) utilizes a real-time tracking system that provides updated prostate position information and allows for correction of the therapeutic beam during treatment with high accuracy. In this study, we evaluate the impact of obesity on patient reported outcomes following SBRT for prostate cancer.

**Materials and methods:**

Between February 2008 and April 2012, 88 obese and 178 non-obese patients with PCa were treated with SBRT at Georgetown University Hospital, Washington, DC. Health-related quality of life (HRQol) was assessed via the expanded prostate cancer index composite (EPIC)-26 at baseline, 6, 12, 18, and 24 months after 5-fraction delivery of 35-36.25 Gy with the CyberKnife. Patients who received androgen deprivation therapy (ADT) were excluded from this analysis due to its known negative impact on HRQoL.

**Results:**

Pretreatment characteristics of obese and non-obese patient groups were similar except that obese patients had lower total testosterone levels. Urinary and bowel function and bother scores between the two patient cohorts were comparable at baseline and subsequent follow-ups. Sexual function and bother were also similar at baseline between both groups. Bother was defined by displeasure patients may experience from functional decline. At 24 months post-SBRT, obese men experienced borderline clinically significant decrease in sexual function and greater sexual bother compared to non-obese patients. Fatigue was significantly higher in obese patients compared to non-obese patients at 18 months post-SBRT.

**Conclusions:**

Prostate SBRT affects obese and non-obese patients similarly in total HRQoL scores and majority of its domains. Obesity has been associated with cancer recurrence; therefore longer follow-up is required to determine the impact of obesity on cancer control.

## Introduction

Obesity (Body Mass Index (BMI) > 30 kg/m^2^) and associated poor health-related quality of life (HRQoL) affect nearly one-third of American men over 60 years old [1]. HRQoL in cancer patients is dynamic and may be adversely impacted by obesity. Excess abdominal fat is associated with an increased risk of urinary incontinence and sexual dysfunction [2]. Total serum testosterone levels are inversely associated with BMI [3], and low levels may contribute to greater incidences of fatigue in obese patients [4].

In the United States, approximately 220,000 men are newly diagnosed with prostate cancer (PCa) each year [5]. Obesity may have a multifaceted impact on a PCa diagnosis and management [6]. Men with a high BMI may have greater incidences of aggressive PCa [7]. Due to inherent technical difficulties associated with increased abdominal adipose tissue distribution, such as setup inconsistencies and increased prostatic movement during treatment [8], obesity can have a negative impact on PCa radiation therapy (RT) outcomes, with obese patients experiencing higher rates of biochemical recurrence and PCa specific mortality [9]. Cancer control outcomes following brachytherapy are not affected by obesity presumably due to the image-guided placement of radioactive sources directly within the prostate [10-11]. Obesity may also have a negative impact on post-RT HRQoL, due to greater radiation exposure to the rectum, bladder, and sexual organs [12-13].

Robotic stereotactic body radiation therapy (SBRT) involves conformal dose delivery of a few hundred, non-coplanar treatment beams from a linear accelerator mounted on a flexible robotic arm. It employs real-time image guidance to track implanted fiducials in the prostate, accounting for prostatic movements in six dimensions [14]. This allows delivery of the therapeutic beam to the prostate with less than 1 mm error, potentially minimizing the volume of critical structures receiving radiation [15]. Increased treatment accuracy in obese patients may further reduce critical organ scatter and ultimately improve HRQoL. This study reports HRQoL in obese men after SBRT for PCa by examining the relationship between BMI and commonly associated urinary symptoms, bowel symptoms, sexual function, and hormonal symptoms after SBRT.

## Materials and methods

### Patient selection

Patients eligible for study inclusion had clinically localized PCa treated with SBRT at Georgetown University Hospital. Patients who received androgen deprivation therapy (ADT) were excluded from this analysis due to its known negative impact on HRQoL [16]. This retrospective review was approved by the Georgetown University Internal Review Board (IRB 2009-510). Patient BMIs were calculated from the baseline weight and height [17]. Obesity was defined as a BMI ­>30 kg/m^2 ^[18-19]. PCa risk groups were defined using the D'Amico criteria [20]. Other patient and treatment characteristics such as age, race, Charleson comorbidity index (CCI) [21], prostate volume, pretreatment prostate-specific antigen (PSA) and testosterone, Gleason score, use of sexual aid, and SBRT dose were acquired from the medical records.

### SBRT treatment planning and delivery

Accuray’s CyberKnife was employed to treat the prostate as previously described [22]. Treatment planning involved fusion of thin-cut CT images and high-resolution MR images, after 4-6 gold fiducials were placed in the prostate. The clinical target volume (CTV) included the prostate and proximal seminal vesicles. The planning target volume (PTV) was defined as the CTV and 3 mm in the posterior direction and 5 mm in all other directions. The rectum, bladder, testes, and penile bulb were contoured and further evaluated with dose-volume histogram (DVH) analysis, using multiplan inverse treatment planning. The PTV received 35-36.25 Gy in five fractions of 7-7.25 Gy over one to two weeks.

### Follow-up and statistical analysis

Patients completed the expanded prostate cancer index composite (EPIC)-26 questionnaire at baseline (one hour prior to first SBRT fraction) and during routine follow-up visit every six months after completion of SBRT, for two years. The EPIC-26 urinary domain was divided into two functional sub-domains, incontinence and irritative/obstructive domains [23]. In addition, one question assessed associated overall bother. The EPIC-26 bowel domain included five questions related to individual symptoms and one question related to overall bother. The EPIC-26 sexual function domain utilized five questions regarding sexual function and one question regarding sexual bother. Lastly, the EPIC-26 hormonal domain had five questions with one item assessing lack of energy.

EPIC scores for each domain and the individual questions ranged from 0-100, with lower values representing worsening symptoms. To statistically compare responses between the two BMI groups, the responses were assigned a score, and the significance of the scores was assessed using Mann-Whitney U test. Clinically significant change was assessed by the minimally important difference (MID) in the EPIC score. This was defined as a change of one-half standard deviation (SD) from the baseline [24]. The Wilcoxon signed-rank test was conducted to determine the statistical significance of the average score of the cohort at each time point.

## Results

Between February 2008 and April 2012, 88 obese and 178 non-obese prostate cancer patients were treated on an institutional SBRT protocol. Characteristics of both obese and non-obese groups were similar prior to SBRT, with a few important differences (Table 1). The median patient age was 68 years for obese and 70 years for non-obese. The obese and non-obese cohorts were composed of 56.8% and 55.6% Caucasian, 41.0% and 36.0% African ancestry, respectively. The median prostate volume in both groups was 38 cc. Pre-treatment PSA values were similar, but baseline pre-treatment total serum testosterone levels varied; 360.5 ng/dL in non-obese and 265.5 ng/dL in obese patients. Additionally, significant comorbidities were more common in obese patients. The D’Amico classification shows a majority, 60.9% of obese and 52.5% of non-obese patients, were intermediate-risk. Seventy-eight percent of both cohorts were treated with 36.25 Gy in five 7.25 Gy fractions.

**Table 1:** Patient Baseline Characteristics *PSA = *Prostate Specific Antigen *SBRT = *Stereotactic Body Radiation Therapy

		All (N =266)	Non-obese (N= 178)	Obese (N= 88)
Age (Years)	Median Age (Range)	69 (44-94)	70	68
Race	White	56.0%	55.6%	56.8%
	Black	37.6%	36.0%	41.0%
	Other	6.4%	8.4%	2.3%
Charlson Comorbidity Index	CCI=0	66.9%	73.0%	54.6%
	CCI=1	22.6%	16.3%	35.2%
	CCI>2	10.5%	10.7%	10.2%
Body Mass Index (kg/m2)	18.5- 24.9 (n)	48	27.0%	--
	25-29.9 (n)	130	73.0%	--
	30-34.9 (n)	61	--	69.3%
	35-39.9 (n)	23	--	26.1%
	40.0-44.9 (n)	4	--	4.6%
Prostate Volume (cc)	Median Volume (Range)	38.0 (9.3-138.7)	38 (9.3-138.7)	38 (17.6-86.2)
Pre-Treatment PSA	Median PSA(Range)	6.0 (0.8-32.5)	6.1 (0.8-32.5)	5.8 (1.5-18.6)
Pre-Treatment Testosterone (ng/dL)	Median Testosterone (Range)	320 (71-1149)	360.5 (106-980)	262.5 (71-114)
Risk Groups (D’Amico’s)	Low Risk	39.5%	41.0%	36.4%
	Intermediate Risk	55.3%	52.3%	61.4%
	High Risk	5.3%	6.74%	2.3%
Sexual Aid	None	63.0%	63.3%	62.5%
	Any Aid	37.0%	36.7%	37.5%
SBRT Dose	36.25 Gy	78.2%	78.2%	78.2%
	35 Gy	21.2%	21.2%	20.7%
	Other	0.8%	0.6%	1.2%

The baseline summary scores of both BMI groups were comparable (Table 2). There were no differences in urinary incontinence scores and urinary irritation/obstruction scores between the obese and non-obese cohorts. Urinary bother scores were similar between BMI groups. Bowel function at baseline was 94.44 in obese and 95.02 in non-obese patients, while associated bowel bother was 90.12 and 91.20, respectively. Patients in both BMI groups had low but similar sexual function (*p* = 0.305) and bother (*p* = 0.487). Lastly, summary of hormonal symptoms between BMI groups remained consistent, with 91.55 in obese and 92.49 in non-obese.

**Table 2:** Pre-Treatment Quality of Life (QoL) EPIC-26 scores *EPIC = *Expanded Prostate Cancer Index Composite *Std. Dev* = Standard Deviation

			All (N =266)			Not Obese (N= 178)		Obese (N= 88)		
			Mean	Std. Dev	MID	Mean	Std. Dev	Mean	Std. Dev	*p*-value
Urinary										
	Incontinence		86.31	13.46	6.73	86.25	13.98	86.45	12.40	0.764
	Irritative/Obstructive		87.32	12.61	5.52	86.37	13.47	89.29	10.44	0.129
	Bother		78.10	25.74	12.87	76.40	26.46	81.61	23.95	0.083
Bowel										
	Function		94.83	9.39	4.69	95.02	8.97	94.44	10.24	0.765
	Bother		90.85	19.06	9.53	91.20	18.44	90.12	20.39	0.27
Sexual										
	Function		53.21	32.43	16.22	54.93	31.96	49.67	33.29	0.305
	Bother		64.76	35.83	17.92	66.06	34.91	62.07	37.71	0.487
Hormonal										
	Summary		92.18	11.44	5.72	92.49	10.81	91.55	12.67	0.934

**Figure 1:**
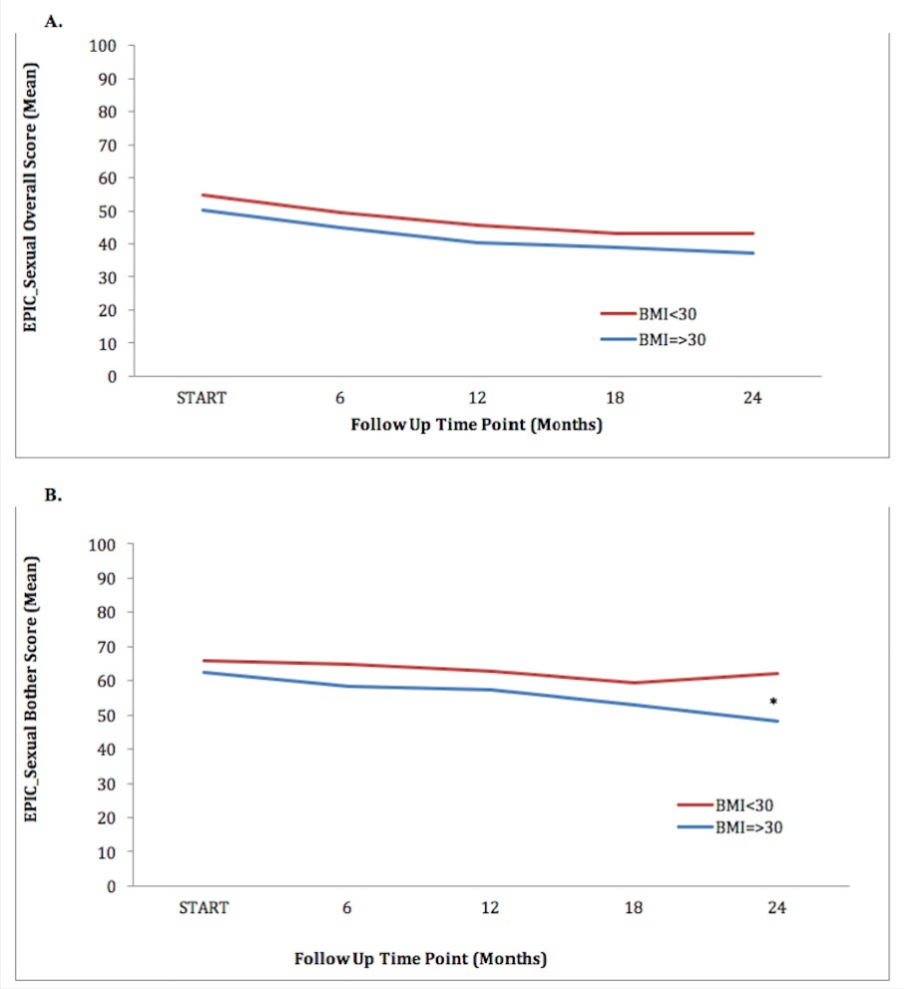
Non-Obese (red) and Obese (blue) Patients Reported Mean Quality of Life (QoL) EPIC-26 Sexual Domain Scores at Baseline and Following SBRT for Prostate Cancer. Shown are plots for: (a) EPIC sexual overall functional summary, (b) EPIC sexual bother.

Table 3 shows the EPIC summary scores linearly from baseline to 24 months after SBRT. Collected scores of urinary incontinence, irritative/obstructive symptoms, and bother were comparable between obese and non-obese groups at all time points. Bowel function and associated bother between the two cohorts were also statistically similar. Summary score of sexual function seems to decline in both obese and non-obese men, but remains similar over the 24 months (Figure 1a). Sexual bother score in obese men (48.05) was significantly lower (p= 0.0076) than that reported by non-obese men (62.26), only at 24 months (Figure 1b). 

**Table 3:** Obese and Non-Obese Patient Urinary, Bowel, Sexual, and Hormonal Domain EPIC-26 Scores *Sexual bother between obese and non-obese patients at 24 months is statistically significant (*p*-value of 0.01). *EPIC = *Expanded Prostate Cancer Index Composite *Std. Dev* = Standard Deviation

		Baseline			6 Month				12 Month				18 Month				24 Month				
		Mean		*p*-value	Mean		*p*-value		Mean		*p*-value		Mean	*p*-value			Mean				*p*-value
		Not Obese	Obese		Not Obese	Obese			Not Obese	Obese			Not Obese	Obese			Not Obese			Obese	
Urinary																					
	Incontinence	86.3	86.5	0.76	90.0	90.3	0.40		87.7	85.7	0.91		85.4	84.7	0.95		86.4			87.2	0.97
	Irritative/ Obstructive	86.4	89.3	0.13	85.8	86.5	0.38		82.8	84.3	0.48		84.7	85.2		0.77			85.9	89.0	0.18
	Bother	76.4	81.6	0.08	77.4	76.6	0.86		68.4	68.7	0.88		75.5	72.0		0.60			75.8	76.6	0.83
Bowel																					
	Function	95.0	94.4	0.77	91.4	90.8	0.42		91.3	90.0	0.73		91.6	91.2	0.41			92.8		93.3	0.70
	Bother	91.2	90.2	0.3	86.7	89.7	0.26		84.4	84.8	0.57		87.2	89.5	0.26			89.2		90.3	0.44
Sexual																					
	Function	54.7	50.1	0.31	49.5	44.8	0.25		45.7	40.4	0.27		43.3	39.1	0.38			43.2		37.2	0.17
	Bother	65.9	62.5	0.49	64.8	58.2	0.16		62.7	57.3	0.28		59.5	53.0	0.20			62.3		48.1	0.01*
Hormonal																					
	Function	92.4	91.7	0.93	92.3	89.6	0.14		91.8	88.4	0.08		92.5	88.5	0.07			91.6		89.8	0.41

**Figure 2:**
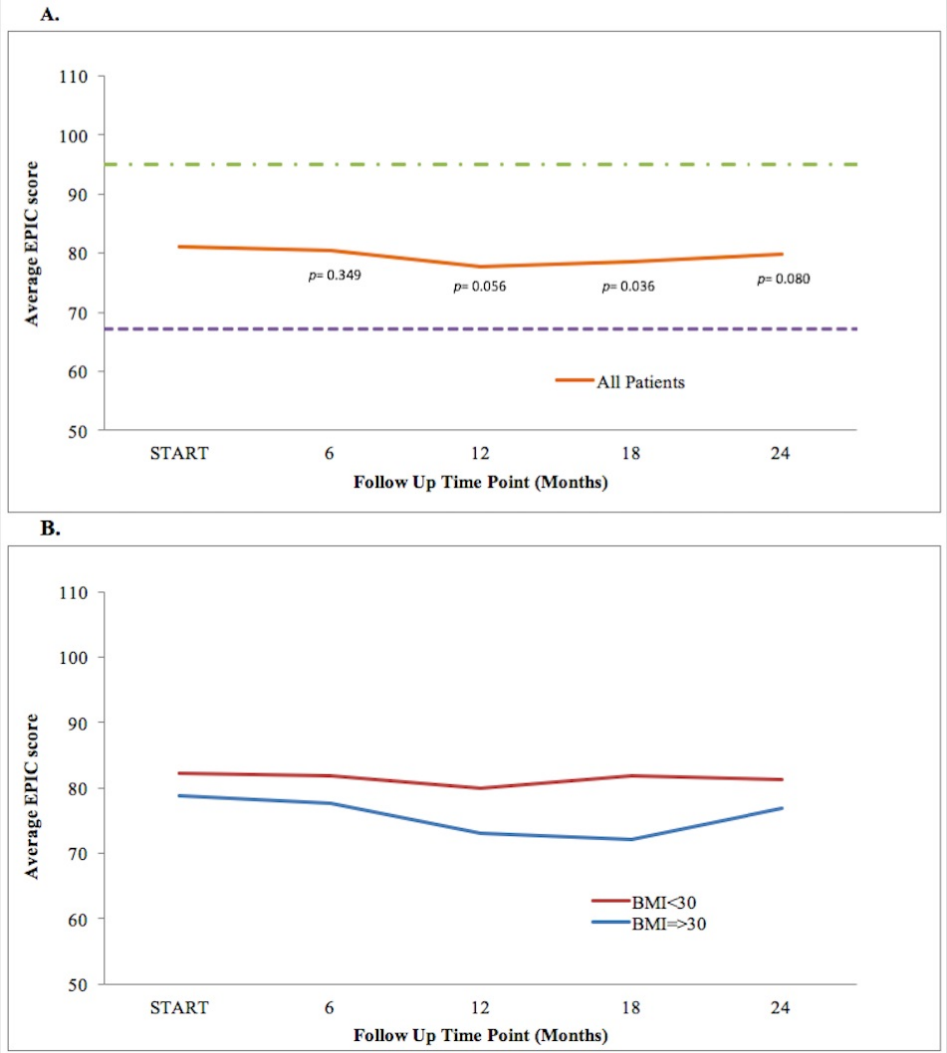
EPIC-26 Loss of Energy Scores Shown is plot for: (a) EPIC loss of energy score at baseline and following SBRT for prostate cancer, (b) EPIC loss of energy score in obese (blue) and non-obese (red) patients with prostate cancer. The thresholds for clinically significant changes in scores (½ standard deviation above, *green;* and below the baseline, *purple*) are marked with dashed lines. EPIC scores range from 0–100 with higher values representing a more favorable health-related QoL.

The EPIC-26 inquired on lack of energy after SBRT, at each follow-up. Compared to baseline, all time points except at 18 months had similar fatigue scores as shown in Figure 2a. However, this difference was not clinically insignificant. At 18 months, the average fatigue score of all patients in the study (78.52) was less than that at baseline (81.92) (*p *= 0.036). Fatigue was relatively constant, except at 18 months when it decreased. Lack-of-energy scores were statistically different (p = 0.042) in obese (72.03) and non-obese patients (81.79) at 18 months, as seen in Figure 2b. At other times points, lack-of-energy scores between both BMI groups were similar.

## Discussion

Consideration of urinary, bowel, sexual, and hormonal side effects are critical in an individual patient’s choice of treatment for prostate cancer [12]. Excess abdominal adipose tissue in obese men may hinder the accuracy of therapeutic beams, thereby diminishing their efficacy, and the increased critical organ scattered dose may significantly compromise the patients’ HRQoL. The comparison of urinary and bowel functions and bother post-SBRT between obese and non-obese patients demonstrates only limited differences. Utilization of fiducial markers with inter and intrafraction image guidance may have reduced potential differences in HRQoL between BMI groups. Our results appear similar to obese and non-obese patients having undergone brachytherapy for PCa [11, 25].

Both sexual function and bother are affected by biological, psychological, and sociological factors (e.g., serum testosterone levels, age, confidence, marital status, and partner satisfaction). Sexual function is clinically decreased in obese men and associated bother is greater than that in non-obese men 24 months after SBRT. While radiation may have certainly contributed to declining sexual function in both BMI groups, it is unlikely to have uniquely affected obese patients. A low baseline serum testosterone level in obese men may be a causative factor for increased late sexual dysfunction, bother, and lack of energy.

Fatigue is a common problem in obese patients and may also be a side effect of RT [26]. The specific etiology of RT-related fatigue is poorly understood and most likely multi-factorial. Fatigue levels were similar between obese and non-obese patients at baseline and most follow-ups after SBRT. Although clinically insignificant, obesity seems to play a role in patient-reported fatigue at the 18-month follow-up. Obese men reported greater levels of fatigue compared to non-obese men only at 18 months after SBRT; at all other time points, obesity does not seem to enhance fatigue in a PCa patient following SBRT.

The present study has several limitations. This is a retrospective study of prospectively collected data from a single institution cohort. This limits the translational generalizability to institutions whose patient population and SBRT protocols are not similar. Only a small number of our patients were morbidly obese (BMI >40), limiting our ability to access the impact of morbid obesity on post-SBRT HRQoL. 

## Conclusions

Prostate SBRT affects obese and non-obese patients similarly in a majority of HRQoL domains. Minimal differences in HRQoL were identified between obese and non-obese patients post-SBRT. A longer follow-up is required to determine the impact of obesity on cancer control.
